# Conductive Porous MXene for Bionic, Wearable, and Precise Gesture Motion Sensors

**DOI:** 10.34133/2021/9861467

**Published:** 2021-06-09

**Authors:** Shengshun Duan, Yucheng Lin, Zhehan Wang, Junyi Tang, Yinhui Li, Di Zhu, Jun Wu, Li Tao, Chang-Hwan Choi, Litao Sun, Jun Xia, Lei Wei, Baoping Wang

**Affiliations:** ^1^Joint International Research Laboratory of Information Display and Visualization, School of Electronic Science and Engineering, Southeast University, Nanjing 210096, China; ^2^School of Materials Science and Engineering, Southeast University, Nanjing 211189, China; ^3^Center for 2D Materials, Southeast University, Nanjing 211189, China; ^4^Center for Advanced Materials and Manufacture, Joint Research Institute of Southeast University and Monash University, Suzhou 215123, China; ^5^Department of Mechanical Engineering, Stevens Institute of Technology, Hoboken, New Jersey 07030, USA; ^6^SEU-FEI Nano-Pico Center, Key Laboratory of MEMS of Ministry of Education Collaborative Innovation Center for Micro/Nano Fabrication Device and System, Southeast University, Nanjing 210096, China; ^7^Center for Advanced Carbon Materials, Southeast University and Jiangnan Graphene Research Institute, Changzhou 213100, China

## Abstract

Reliable, wide range, and highly sensitive joint movement monitoring is essential for training activities, human behavior analysis, and human-machine interfaces. Yet, most current motion sensors work on the nano/microcracks induced by the tensile deformation on the convex surface of joints during joint movements, which cannot satisfy requirements of ultrawide detectable angle range, high angle sensitivity, conformability, and consistence under cyclic movements. In nature, scorpions sense small vibrations by allowing for compression strain conversion from external mechanical vibrations through crack-shaped slit sensilla. Here, we demonstrated that ultraconformal sensors based on controlled slit structures, inspired by the geometry of a scorpion's slit sensilla, exhibit high sensitivity (0.45%deg^−1^), ultralow angle detection threshold (~15°), fast response/relaxation times (115/72 ms), wide range (15° ~120°), and durability (over 1000 cycles). Also, a user-friendly, hybrid sign language system has been developed to realize Chinese and American sign language recognition and feedback through video and speech broadcasts, making these conformal motion sensors promising candidates for joint movement monitoring in wearable electronics and robotics technology.

## 1. Introduction

Precise, reliable, and wide range joint movement monitoring including posture acquisition, behavior analysis, etc. is of value in studies of cognitive neuroscience, sports and military training, human-machine interfaces, and remote control [1–14]. To achieve real-time joint movement monitoring, precise and stable flexible hybrid electronic systems that integrate conformal physical sensors with miniaturized silicon-based rigid microcircuit chips can provide consumer-grade performance and reliability [15–28]. Thus, stable, precise conformal motion sensors with high sensitivity in a wide-angle range can not only reduce the computational cost but also improve system reliability and recognition rate. Most previous reported motion sensors focused on utilizing the tensile deformation of the convex surface of joints to monitor joint movements. The nano/microcracks on the sensing layer induced by the tensile deformation during joints bending cause changes in electrical properties [29]. Yet, the cracks are inconsistent and even unrecoverable during thousands of cycles of elastic deformation due to large-scale body movements, thereby leading to data inconsistency or errors. On the other hand, the tensile strain on the convex surface concentrates in the joint and varies unevenly during joint movements, and the joint folds/creases on the convex surface of joints erect obstacles in mechanical conformality and compliance of wearable electronics and in small-angle detection for joint movements [30–32]. Otherwise, it is noting that the concave surfaces of joints come close to each other and induce compression strain during joint movements.

**Figure 1:**
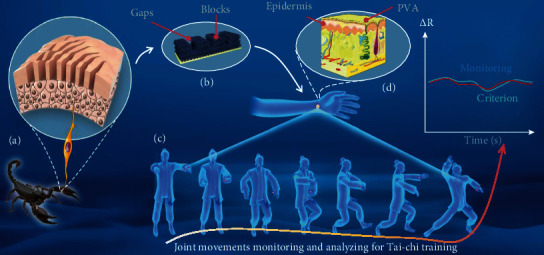
Illustration of the bioinspired sensor and its potential application. (a, b) Schematic of the analogy between the motion sensor and the scorpion. (c) Monitoring of training action for material arts training (Tai-chi). (d) Conformal contact between the motion sensor and the human skin.

Scorpions have crack-shaped slit sensilla that involve parallel, periodic two-body tissue phases [33, 34]. Such slit geometry and contexture enable ultrasensitive displacement detection by allowing for mechanical compliance, which leads to the compression strain of the slit corresponding to small external force variations, so as to effectively detect vibrations in their surroundings [33, 35, 36]. An illustration of the scorpion's slit sensilla is shown in Figure 1(a); the scorpions have strain detectors located near the leg joint between the metatarsus and tarsus bones. Such detectors consist of a viscoelastic pad, with the slit sensilla composed of parallel sensory cracks that coated with soft cuticular membranes embedded in hard blocks periodically, of which the elastic modulus of blocks is one order higher than that of the cuticular membrane [33]. The slit sensilla are directly connected to the nervous system to sense strain displacement thereby efficiently collecting external vibrations.

In this work, inspired by the geometry and principle of the slit sensilla, we design wide range, precise, and conformal motion sensors by transferring parallel, 3 mm thick MXene-coated polyurethane (PU) sensory film onto the top of a viscoelastic polymer, poly(vinyl alcohol) (PVA). Analogous to the crack-shaped slit sensilla, we constructed controlled slit structures in the sensory film through laser engraving techniques, which are composed of parallel, periodic two-body tissue phases involving MXene-coated PU hard blocks and soft air gaps. The electrical conductance across the slit structures is measured. The similarity lies in the slit geometry and contexture, known to be the key to stimulus conversion and slit sensilla ultrasensitivity. The PU and adherent PVA possess high mechanical compliance and elastic restorability thereby endowing motion sensors with remarkably conformal and durable. The slit structures enable conformal motion sensors precise sensation of compression strain on the concave surfaces during joint movement through double contact effect. The motion sensor performances such as sensitivity and minimum angle detection threshold vary with slit ratio (defined as the width ratio of blocks and gaps, Figure 1(b)). The conformal motion sensors with a 1/2 slit ratio exhibit high sensitivity (~0.45%deg^−1^), ultralow angle detection threshold (15°), fast response/relaxation times (115/72 ms), wide range (15°~120°), and durability (over 1000 cycles). Furthermore, the conformal motion sensors were applied at joints, such as the neck, elbow, finger, knee, and ankle, to detect joint movements. Also, we developed a hybrid sign language recognition system, enabling precise recognition of similar gestures and interactive feedback by allowing for video and speech. Furthermore, inspired by the Schmitt trigger [37], a bistable constraint criterion was proposed to eliminate recognition errors caused by voltage jitter and consequently improve recognition rate and system stability.

## 2. Results

### 2.1. Conformal Motion Sensors

As illustrated in Figure 1(a), to mimic the scorpion slit organ that consists of a viscoelastic pad, with the slit sensilla composed of parallel, periodic two-body tissue phases with different elastic modulus, we proposed a conformal motion by transferring a sensory film with slit structures involving parallel, periodic MXene-coated hard PU blocks and soft air gaps onto the top of a viscoelastic, adherent PVA (Figure 1(b)). The conformal motion sensors are attached to the concave surface of joints to monitor joint movements by detecting the bending-induced compression strain through the double contact effect. The as-proposed conformal motion sensors exhibit high sensitivity, wide detection range, and remarkable reliability, which, therefore, can be used to monitor whether training action is standard such as material arts training (Tai-chi), sports training, and rehabilitative training (Figure 1(c)).

**Figure 2:**
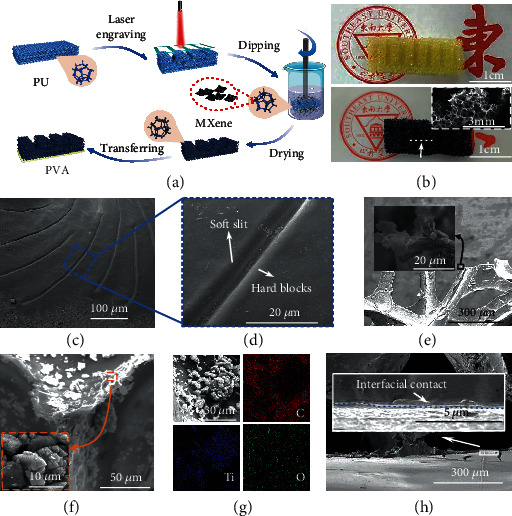
Fabrication and structural characterization of motion sensors. (a) The fabrication process of the motion sensor. (b) Optical images of PU blocks and MXene-coated PU blocks. Inset is an SEM image for MXene-coated PU blocks. (c, d) The SEM image of the slit sensilla of the scorpion. (e) The SEM image of the lateral wall of the blocks. (f) The SEM image of MXene-coated keel structures, showing the uniform distribution and tight interfacial contacts between MXene and PU keel structures. (g) Corresponding energy-dispersive X-ray spectroscopy (EDS) mapping of C, Ti, and O in the MXene-coated PU keel structures. (h) A tight interfacial contact is formed between the PVA gel and the MXene-coated PU blocks. The inset shows the details of the tight interfacial contact.

The sensory film with slit structures was fabricated through laser engraving and multistep dip-coating techniques (details are in “Methods” in “Experiment section”), as shown in Figure 2(a). PU sponge was adopted as the matrix due to its high mechanical compliance, elastic restorability, high cell density, and low cell size [38, 39]. Such cell density and size enable PU matrix large adsorption volume of functional dispersion in each dip-coating process (Supplementary Figure S1) and effective contact area with each other in joint movements. MXene was chosen as an active layer because of its fascinating electrical properties and high specific surface areas [40–44]. Such high specific surface areas improve the binding force of MXene nanosheets with PU matrix, therefore leading to better cycling reliability. The optic and SEM images of the sensory film were illustrated in Figure 2(b), where MXene was uniformly anchored onto the skeleton of the PU matrix to form a 3-dimensional active sensory network. The sensory film is extremely lightweight, about 33.5 mg (Supplementary Figure S2). Similar to the geometry and contexture of scorpion slit sensilla (Figures 2(c) and 2(d)), the alternating soft air gaps and hard MXene-coated PU blocks form a strain detection layer. For MXene-coated PU blocks, we observed the distribution of MXene in external (Figure 2(e)) and inner (Figure 2(f)) PU keel structures, indicating that the uniform and compact distribution and tight interfacial contacts between MXene and PU keel structures are formed. The corresponding energy-dispersive X-ray spectroscopy (EDS) mapping results for C, Ti, and O elements furtherly verify the existence and compact distribution of MXene coated in PU keel structures (Figure 2(g)). Such uniform distribution contributes to the enhanced conductivity and cycling stability of sensory films.

The sensory film was then transferred onto the PVA gel (Figure 2(a) and Supplementary Figure S3) to prepare conformal motion sensors. The PVA gel in our experiments came from the nose cream, a kind of skincare product whose main compositions are water, PVA, and acrylic ester, which are self-adhesive, biocompatible, and biodegradable at room temperature [14, 45, 46] and are, thus, adopted as the viscoelastic substrate. The biodegradability is depicted in Supplementary Figure S4 where the PVA is totally degraded in water at room temperature, thereby enabling motion sensors' complete removal from the skin using water, without any residues. Also, due to the similar stretchability with human skin and self-adhesion, PVA gel forms formal interfaces with both sensory film (Figure 2(h)) and human skin (Figure 1(d)). Therefore, the PVA gel peeled from the finger joint retained the entire texture information of the skin (Supplementary Figure S5). Yet, the poor breathability of PVA partly influences the wearable comfortability of our motion sensors.

### 2.2. Working Mechanism and Electrical Characteristics of Conformal Motion Sensors

**Figure 3:**
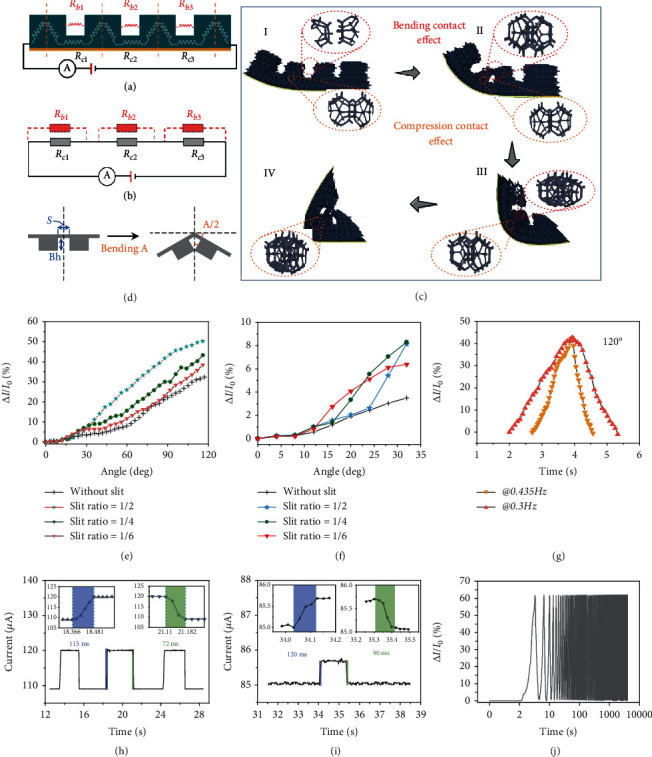
Working mechanism and electrical performance of motion sensors. (a) The equivalent resistance model of the as-prepared sensor. (b) The equivalent circuit diagram of the motion sensor. (c) The schematic diagram of compression strain in the motion sensor during bending. (d) The mathematic model shows the minimal angle where the adjacent blocks connect with each other. (e) The relative changes of electrical conductance for sensors with different slit ratios when the bending angle increases from 0 to 120°. (f) The electrical conductance of sensors with different slit ratios when the bending angle only increases from 0 to 30°. (g) The current-angle response of the sensor exhibits bending-rate-independent sensing behavior. (h) The response/relaxation times of the motion sensor with 1/2 slit ratio when bent from 90° to 120°. (i) The response/relaxation times of the motion sensor with 1/4 slit ratio when bent from 0° to 30°. (j) The durability test under continuous bending–unbending cycles at 120°.

To further analyze the working mechanism of the motion sensor, an equivalent resistance model of this motion sensor was established. The whole resistance includes bulk resistance (*R*_*c*_) of MXene-coated PU hard blocks and interfacial resistance (*R*_*b*_, infinite before the adjacent blocks contact each other) of adjacent PU-Mxene blocks, as shown in Figure 3(a). The corresponding electro circuit is illustrated in Figure 3(b). As seen, the bulk resistance (*R*_*cx*_, *x* = 1, 2, 3) and interfacial resistance (*R*_*bx*_, *x* = 1, 2, 3) are parallel, respectively. The resistance-change model of *R*_*bx*_ is depicted in Figure 3(c), where the emergence of interfacial resistance is at about 2arctan(*S*/2Bh) (*S* is the length of slits, and Bh is the height of blocks). The working principle of the motion sensor was shown in Figure 3(c). During joint bending, the contact among internal MXene-coated PU keel structures (denoted as compression contact effect) and the contact between adjacent MXene-coated PU blocks (denoted as bending contact effect) increase. Such contacts can increase conductive pathways and then increase electrical conductance, thereby decreasing the bulk resistance (*R*_*c*_) and interfacial resistance (*R*_*b*_) (Supplementary Figure S6). It is noting that the two effects which one mainly contributes to electrical conductance changes may vary in different bending phases (Figure 3c).

The slit ratio may affect the performances of motion sensors, like sensitivity and minimum angle detection threshold. Therefore, we prepared four motion sensors with slit ratios of 0, 1/6, 1/4, and 1/2, respectively, of which the structural parameters and initial resistance are depicted in Supplementary Figure S7. The initial resistance varies with the length of the sensor linearly (Supplementary Figure S8). The as-prepared motion sensors were placed on homemade testing equipment (Supplementary Figure S9) with its two electrodes connected to the source meter to record electrical conductance. As the bending angle increases, a sharp and wide range electrical conductance change of motion sensors with a 1/2 slit ratio is observed, yet the rest motion sensors exhibit similar angle-sensing properties (Supplementary Figure S10). Such phenomena are most possibly related to compression strain differentiation in sensors with different slit ratios. The bending angles where the contact effect (interfacial resistances) disappears relate to the slit ratio of sensors. For slit-based sensors, smaller slit ratio leads to disappearance of bending contact effect at smaller bending angles and only the compression contact effect contributes to the increasement of electrical conductance as the bending angle continues to increase, thereby leading to similar electrical conductance changes with sensors without slits after the bending angles reach to some certain values. Besides, as depicted in Figure 3(e), for motion sensors without slit structure, the internal contact area rapidly increases after a certain bending angle (about 60°), so that the relative change of electrical conductance (Δ*I*/*I*_0_) curve shows typical quadratic characteristics and lower sensitivity at smaller angles. In contrast, for motion sensors with slit structure, the introduction of soft air gaps on the PU film reduces the internal contact areas and increases the bending contact effect between adjacent blocks. Therefore, the sensitivity, denoted as Δ*I*/(*I*_0_ · angle), of motion sensors with a 1/2 slit ratio is 2 times higher than that of sensors without microstructure (Figure 3(e)).

Furtherly, we explored the sensing ability of the four motion sensors at a small bending angle (~30°). More narrow gaps enable adjacent blocks to contact each other at smaller bending angles. Therefore, the motion sensors with 1/6 and 1/4 slit ratios exhibit higher sensitivity at small angles, yet the motion sensor of 1/2 slit ratio cannot detect bending angles lower than 10° (Figure 3(f)). Furthermore, as shown in Figure 3(d), the minimal detectable angle can be estimated by 2arctan(*S*/2Bh). Therefore, the recorded and calculated minimal angles are depicted in Supplementary Table S1, exhibiting good consistency. Then, the sensor is bent from 0 to 90° at two different frequencies (0.435 Hz and 0.3 Hz). As depicted in Figure 3(g), the current-angle response of our sensors exhibits bending-rate-independent sensing behavior. Moreover, we tested the response time of conformal motion sensors when the bending angle increased from 90° to 120° (Figure 3(h)). The response/relaxation times, denoted as the time motion sensors take to reach their final signal amplitude/the time motion sensors take to return to their original signal once the stimulus is removed [47], are 115/72 ms, which is enough for most practical applications such as dynamic posture acquisition. The response/relaxation times of the motion sensor with 1/4 slit ratio when the bending angle increased from 0° to 30°, as shown in Figure 3(i), are 120/90 ms, exhibiting similar response characteristics with that of sensors with 1/2 ratio at large bending angles. The motion sensor exhibits enhanced stability for the static bending angle, a key index to precisely obtain the static posture information. To test the stability and repeatability for long-term service, we also performed over 1000 bending–unbending cycles (at the bending angle of 120°), and the current profiles showed no obvious degradation (Figure 3(j)).

### 2.3. Optical Feedback for Joint Bending Angles

**Figure 4:**
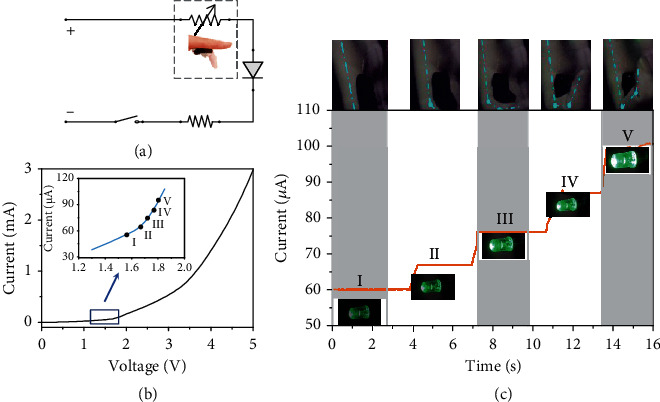
Optical feedback for finger motions. (a) Circuit diagram of the optical feedback system. (b) Voltage–current characteristics of the green LED with 0.06 W rated power. (c) The brightness of the green LED gradually increases with the bending angle.

Optical feedback for gestures plays a crucial role in human-machine interactive systems. Thus, a visual feedback system for joint bending angles was designed as shown in Figure 4(a). When the bending angle gradually increases, the green light-emitting diode (LED) becomes brighter, exhibiting excellent optical feedback performance. As shown in Figure 4(a), a commercial green LED, whose brightness increases with power, was serially connected with the sensor under an input voltage of 15 V. Figure 4(b) displays the voltage–current characteristic curve of the green LED operated at 0.06 W rated power, and the inset indicates the working region of the LED in this designed experiment. The motion sensor can be used as a rheostat whose resistance varies with the bending angle. Finger bending can increase the current flowing through the green LED by decreasing sensor resistance, thereby enabling the LED more brightness. As depicted in Figure 4(c), the brightness of the green LED gradually increases as the index finger bends.

### 2.4. Gesture Information Acquisition

**Figure 5:**
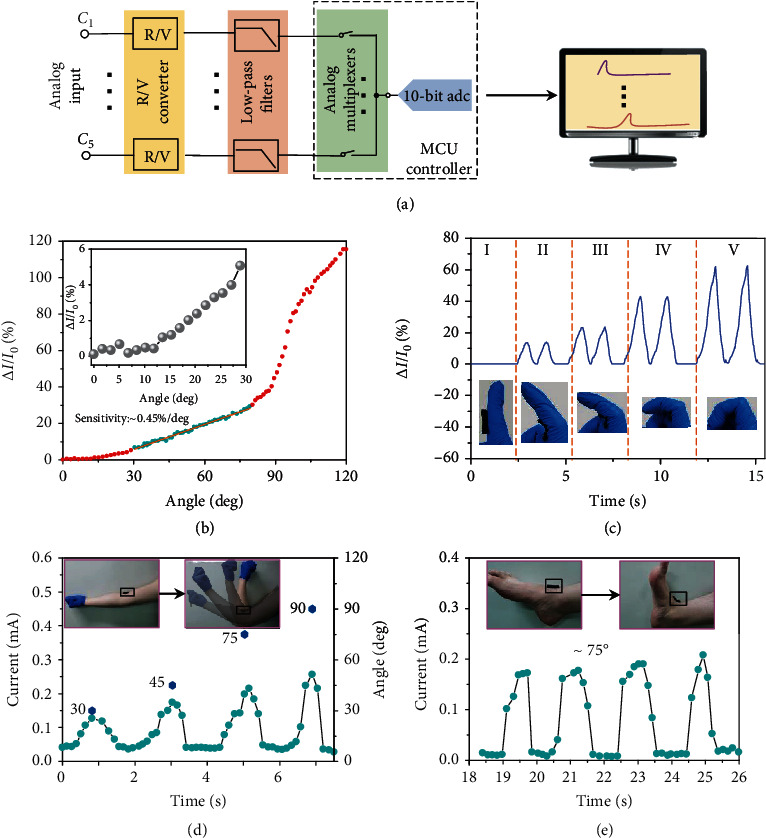
Real-time gesture information acquisition. (a) Circuit diagram showing the signal flow in the real-time gesture information acquisition system, from the acquired analog signals to the digital signals. (b) The current-time curve as the index finger bends, with a distinct linear region. The inset indicates the current changes in small bending angles (<30°). (c) Dependence of the acquisition current signals on index finger motion statuses. The insets, labeled I, II, III, IV, and V, show photographs of the five bending statuses. (d, e) Acquired current signals from (d) elbow bending and (e) ankle bending.

To demonstrate the capability of conformal motion sensors for gesture acquisition, a preliminary step was taken to evaluate the performance of the functional sensing unit in response to finger motions. As shown in Figure 5(a), all analog signals generated from motion sensors first went through signal processing to be converted into digital signals before being transmitted to the display applications. To quantify the angle-sensing properties of conformal motion sensors with a 1/2 slit ratio when attached to joints, the current-angle measurements are conducted after attached to the index finger, and their relationship is plotted in Figure 5(b). The relative change of electrical conductance (Δ*I*/*I*_0_) increases slowly at first and then increases sharply when the angle is larger than 90°, which is ascribed to the drastic squeeze between MXene-coated PU keel structures. In this case, the PU blocks fully in contact with each other and the bending contact effect does not contribute to electrical conductance incensement (Supplementary Figure S11). Also, a linear region is observed for the angle between 30 and 80, and the SA, defines as SA = Δ*I*/(*I*_0_ · angle), is as high as 0.45%deg^−1^. The inset in Figure 5b indicates the minimum angle detection threshold is about 15°, which is possibly ascribe to the rough crease structures at the concave surfaces of joints. Such performances involving sensitivity, response time, detectable angle range, minimum angle detection threshold, and durability are comparable or even higher than most of the reported representative motion sensors (Supplementary Table S2). Figure 5(c) shows the acquired electrical conductance from a conformal motion sensor attached to the index finger corresponding to five different motion statuses: I, II, III, IV, and V (insets, Figure 5(c)). The output reliability of the motion sensor was confirmed by continuous repetition of each finger motion, two times for each status, which resulted in two current peaks in the acquired current signals. Therefore, each motion status of the index finger could be reliably expressed as electric signals from the motion sensor. Besides, the motion sensor can conformally fit on a person's joints such as the wrist, elbow, and ankle to sensitively convert body gestures into electric signals. A subject was asked to wear the motion sensor against the elbow, as shown in Figure 5(d), and characteristic electrical conductance is generated in response to different bending angles (30°, 45°, 75°, and 90°) of the elbow. In addition, the sensor was fixed on the ankle, and the corresponding electrical conductance is measured at about 75° bending angle, as shown in Figure 5(c). Also, the electrical conductance in response to various joint movements like wrist, knee, neck, and throat is illustrated in Supplementary Figure S12.

### 2.5. Precise Recognition and Feedback for Sign Language Gestures

**Figure 6:**
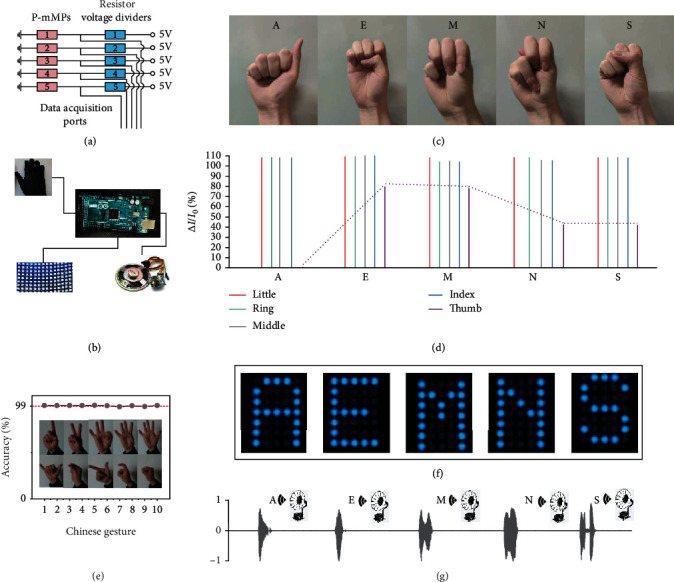
Demonstration of the human-machine interaction. (a) The voltage dividing circuit. (b) Schematic of the sign language recognition and feedback system. (c) Photographs of the sign language hand gestures (A, E, M, N, and S) according to American Sign Language. (d) The corresponding voltage profiles. (e) A high recognition rate was obtained for Chinese gestures. (f) Photographs showing that the gestures were translated into images and displayed through the LED lattice screen. (g) Sound signal for speech playing. The amplitude was normalized.

Owing to the soft and flexible characteristics of the PVA and PU sponge, motion sensors can be comfortably and conformally attached to human joints. Supplementary Figure S13 provides an overview of the process flow of both hardware and software, beginning with an analog signal acquisition, followed by data processing, and finally transmission to a customized feedback application, which is embedded with a robust bistable constraint criterion and a video/voice feedback module. The analog signal for each path is the voltage across the voltage dividing resistor (the voltage dividing circuit is shown in Figure 6(a)). The data processing and transmission path for each sensor are implemented concerning the corresponding transduced signals with an analog circuit, which can effectively remove interference signals and environmental noise using low-pass filters, thereby ensuring that the final analog output of the motion sensor can precisely express the gesture information and is suitable for subsequent processing by an analog-to-digital converter. The feedback application was developed with a built-in multithreshold encoding algorithm, which can code digital signals corresponding to different gestures into a unique binary code. The binary code can then be decoded into the corresponding gesture by traversing the lookup table. Finally, interactive feedback of recognition results can be obtained through video and voice broadcasts. This system consists of a wearable sensor array, an embedded core unit for somatosensory information monitoring and gesture recognition, and an interactive feedback module with video and voice broadcasts (Figure 6(b)). The whole human-machine interface system was presented in Supplementary Figure S14.

To demonstrate precise, real-time gesture recognition, five similar gestures (A, E, M, N, and S, see Figure 6(c)) were selected from American Sign Language. The corresponding current profiles of these gestures are shown in Figure 6(d). Importantly, the gestures we selected are almost identical, with a little difference. Even in this case, the motion sensor exhibits good recognition results owing to its high sensitivity and sensory array. For example, the bending angles of the middle finger of gestures “E,” “N,” and “S” are slightly larger than that of the middle finger of gestures “A” and “M.” Therefore, the current profiles of the middle finger of gestures “E,” “N,” and “S” are larger than that of the middle finger of gestures “A” and “M.” Furtherly, we adopted different methods to improve system stability from hardware design and software algorithm. The low-pass filters are selected to remove interference signals and environmental noise in hardware design. Besides, normal individuals often occur tremors subconsciously in association with excessive physical exertion, excitement, hunger, fatigue, or other causes often called physiological tremors. It will cause voltage jitter across the sensor and encoding errors. Therefore, we proposed a robust bistable constraint criterion to effectively eliminate this negative effect and improve system stability (more comparative analysis is shown in Supplementary Figure S15). To further validate the performance of our human-machine interaction system on the sign language of different countries, we performed a sign language recognition test on Chinese gestures (from one to ten), and a high recognition rate of over 99% was obtained (Figure 6(e)).

A video and voice feedback application with the capability to convert text and image for speech playing and image display was developed to feedback what the machine has learned. As shown in Figures 6(f) and 6(g), the gestures for “A,” “E,” “M,” “N,” and “S” were successfully translated into speech and images. The speech and images were played via the text-to-speech chip and loudspeaker (Supplementary Figure S16) and displayed via the LED lattice screen (Supplementary Figure S17), respectively. The related video can be found in Movie 1. Overall, this work is expected to use a creative bioinspired motion sensor technology to promote the development of user-friendly human-machine interfaces.

## 3. Discussion

We mimicked the crack-shaped scorpion slit sensilla to design a conformal motion sensor by transferring slit-shaped MXene-coated PU blocks on the top of adherent PVA gel. Compared to reported body gesture monitoring sensors applied on convex surfaces, the motion sensor, fixed at the concave surface of joints, could completely utilize the compression strain during joint bending because of the elaborate slit structure, which exhibits high sensitivity (0.45%deg^−1^), ultralow angle detection threshold (~15°), fast response/relaxation times (115/72 ms), wide range (15°~120°), and durability (over 1000 cycles). Furthermore, we developed a user-friendly, hybrid sign language system to realize Chinese and American sign language recognition and feedback through video and speech broadcasts. Furthermore, we developed a bistable encoding algorithm that can eliminate recognition errors by allowing for tolerance of voltage jitter caused by physiological tremor, thereby improving the recognition rate and system stability. This work provides a basis for deeper human-machine interaction such as sign language recognition and complex body language recognition.

Also, more proper materials and precise nano/microengineering of controlled cracks on the scale of nanometers other than the slit structures on the millimeter scale that we prepare here may further improve the performance of our crack-based motion sensors.

## 4. Materials and Methods

### 4.1. Materials

Analytical grades of HCl and LiF were purchased from Sinopharm Chemical Reagent Co., Ltd. MAX (Ti3AlC2, 400 mesh) was purchased from 11 Technology Co., Ltd. Polyurethane sponge was purchased from 3M China. The nose film (Oilyoung, China) mainly consists of water, poly(vinyl alcohol), and acrylic ester (a safety thickening agent). All reagents were used as received without further purification unless otherwise specified.

### 4.2. Preparation of Multilayered Ti_3_C_2_T_x_ Flakes

Powders of (1.0 g) Ti_3_AlC_2_Ti_3_AlC_2_ were added to 20 mL of 6 M HCl solution with 10 g LiF to selectively etch the Al contained in the Ti_3_AlC_2_ MAX raw powders. Wet etching was performed at 35°C with magnetic stirring at 450 rpm for 24 h to obtain a stable suspension. The suspension was then centrifuged at 5000 rpm for 10 min, followed by washing with deionized water several times until the pH of the discarded upper liquid reached 6. The multilayered Ti_3_C_2_T_x_ powders were then obtained after drying on a hotplate at 60°C. The obtained multilayered Ti_3_C_2_T_x_ powders were dispersed in 50 mL deionized water and delaminated by ultrasonication for 1 h. Then, the unexploited multilayered Ti_3_C_2_T_x_ powders were removed by centrifugation at 4000 rpm for 5 min. Finally, few-layered Ti_3_C_2_T_x_ powders were obtained after drying at 60°C.

### 4.3. Preparation of Motion Sensors

The PU sponge was cut into cuboids (2 cm × 0.5 cm × 0.3 cm) and then patterned to have multiple blocks and slits. The numbers of slits and blocks are 3 and 4, respectively. The depth of the slits was 2 mm. The widths of the blocks and slits were varied to prepare and test for various slit ratios. The PU sponge cuboids with slit structures were cleaned repeatedly with deionized water and ethyl alcohol and were then dried on a hotplate. Next, the obtained few-layered Ti_3_C_2_T_x_ powders of 0.15 g were added to 50 mL deionized water, which was stirred at 50°C and 400 rpm for 30 min using a magnetic stirrer, followed by ultrasonication for 10 min to prepare 3 mg/ml MXene aqueous dispersion. Third, the PU sponge cuboids with slit structures were coated with MXene through a multistep dip-coating process. In each step, the cuboids were dipped into the as-prepared MXene aqueous dispersion and squeezed several times to completely absorb the dispersion and were dried on a hotplate at 100°C for 30 min before the next dipping-drying cycle. After the coating cycles were completed, they were placed onto the PVA gel film, which was scraped onto the polydimethylsiloxane (PDMS) substrate uniformly. It was then curdled at room temperature for 20 min. The PU sponge with PVA thin film was then peeled off from the PDMS substrate. The motion sensors were obtained after fixing the Cu wires at the two ends of the PU sponge as conductive electrodes.

### 4.4. Device Characterization

The structure and morphology of the motion sensor were observed and recorded using an SEM (Quanta 200 FEI). The electrical characterization of each flexible motion sensor was performed using a source meter (Keithley 2400) under the signal of 20 V at 100 Hz. A force gauge and homemade angle testing equipment were used to measure the applied force and angle, respectively. All measurements were performed under ambient conditions.

## Data Availability

Supplementary materials contain additional data needed to evaluate the conclusions of the paper. All other data used to support the findings of this study are available from the corresponding author upon request.

## Supplementary Materials

Supplementary Materials Figure S1: before and after the one-time dip coating of the PU sponge in the MXene solution. Figure S2: the obtained MXene-coated PU sponge is extremely lightweight, about 33.5 mg. Figure S3: the one-step transfer technique to prepare the motion sensor. Figure S4: the dissolution of the PVA film in deionized water at room temperature. Figure S5: conformal contact between the PVA and the skin. Figure S6: electrical conductance increases after the two MXene-coated PU blocks contact with each other. Figure S7: the width of blocks and slits of sensors with different slit ratios and their initial resistances. Figure S8: a linear relationship between the initial resistance and sensor length is observed. Figure S9: the schematic illustration of the predesigned testing equipment. Figure S10: the electrical conductance of sensors with different slit ratios when the bending angle increases from 0 to 120°. Figure S11: photograph of motion sensors when bending angle is up to 90°. Figure S12: responses of motion sensors during different joint movements involving wrist, throat, knee, and neck. Figure S13: schematic overview of the hybrid sign language recognition with both hardware and software. Figure S15: comparison of the bistable constraint criterion and single threshold criterion. Figure S16: hardware for the speech module in the circuit diagram. Figure S17: hardware for the display module in the circuit diagram. Table S1: comparison of calculated and recorded minimal angle of motion sensors with different slit ratios. Table S2: comparison of various motion devices and representative device figures of merit. Movie S1: illustration of the hybrid sign language recognition with interactive feedback.
