# Supplementary material for: Conductive Porous MXene for Bionic, Wearable, and Precise Gesture Motion Sensors
Source: Research (Wash D C). 2021 Jun 9;2021:9861467. doi: 10.34133/2021/9861467 (PMC8212815; doi:10.34133/2021/9861467)
Supplement: Supplementary Materials — Figure S1: before and after the one-time dip coating of the PU sponge in the MXene solution. Figure S2: the obtained MXene-coated PU sponge is extremely lightweight, about 33.5 mg. Figure S3: the one-step transfer technique to prepare the motion sensor. Figure S4: the dissolution of the PVA film in deionized water at room temperature. Figure S5: conformal contact between the PVA and the skin. Figure S6: electrical conductance increases after the two MXene-coated PU blocks contact with each other. Figure S7: the width of blocks and slits of sensors with different slit ratios and their initial resistances. Figure S8: a linear relationship between the initial resistance and sensor length is observed. Figure S9: the schematic illustration of the predesigned testing equipment. Figure S10: the electrical conductance of sensors with different slit ratios when the bending angle increases from 0 to 120°. Figure S11: photograph of motion sensors when bending angle is up to 90°. Figure S12: responses of motion sensors during different joint movements involving wrist, throat, knee, and neck. Figure S13: schematic overview of the hybrid sign language recognition with both hardware and software. Figure S15: comparison of the bistable constraint criterion and single threshold criterion. Figure S16: hardware for the speech module in the circuit diagram. Figure S17: hardware for the display module in the circuit diagram. Table S1: comparison of calculated and recorded minimal angle of motion sensors with different slit ratios. Table S2: comparison of various motion devices and representative device figures of merit. Movie S1: illustration of the hybrid sign language recognition with interactive feedback. [file 9861467.f1.zip › 20210517SI.docx]

**Supporting Information**

**Conductive Porous MXene for Bionic, Wearable, and Precise Gesture Motion Sensors**

**Authours**

Shengshun Duan^1^, Yucheng Lin^1^, Zhehan Wang^2,3^, Junyi Tang^1^, Yinhui Li^1^, Di Zhu^1^,

Jun Wu^1*^, Li Tao^2,3,6*^, Chang-Hwan Choi^4^, Litao Sun^3,5,6,7*^, Jun Xia^1^, Lei Wei^1^,

Baoping Wang^1^

**Affiliations**

^1^Joint International Research Laboratory of Information Display and Visualization, School of Electronic Science and Engineering, Southeast University, Nanjing 210096, China

^2^School of Materials Science and Engineering, Southeast University, Nanjing 211189, China；

^3^Center for 2D Materials, Southeast University, Nanjing 211189, China

^4^Department of Mechanical Engineering, Stevens Institute of Technology, Hoboken, New Jersey 07030, USA

^5^SEU-FEI Nano-Pico Center, Key Laboratory of MEMS of Ministry of Education Collaborative Innovation Center for Micro/Nano Fabrication Device and System, Southeast University, Nanjing 210096, China

^6^Center for Advanced Materials and Manufacture, Joint Research Institute of Southeast University and Monash University, Suzhou 215123, China

^7^Center for Advanced Carbon Materials, Southeast University and Jiangnan Graphene Research Institute, Changzhou 213100, China

*corresponding author: [wujunseu@seu.edu.cn](mailto:wujunseu@seu.edu.cn), tao@seu.edu.cn, and slt@seu.edu.cn

Figure S1. Before and after the one-time dip coating of the PU sponge in the MXene

solution.

Figure S2. The obtained MXene-coated PU sponge is extremely lightweight, about 33.5 mg.

Figure S3. The one-step transfer technique to prepare the motion sensor.

Figure S4. The dissolution of the PVA film in deionized water at room temperature.

Figure S5. Conformal contact between the PVA and the skin.

Figure S6. Electrical conductance increases after the two MXene-coated PU blocks contact

with each other.

Figure S7. The width of blocks and slits of sensors with different slit ratios and their initial resistances.

Figure S8. A linear relationship between the initial resistance and sensor length is observed.

Figure S9. The schematic illustration of the pre-designed testing equipment.

Figure S10. The electrical conductance of sensors with different slit ratios when the

bending angle increases from 0 to 120°.

Figure S11. Photograph of motion sensors when bending angle is up to 90°.

Figure S12. Responses of motion sensors during different joint movements involving

wrist, throat, knee, neck.

Figure S13. Schematic overview of the hybrid sign language recognition with both

hardware and software.

Figure S15. Comparison of the bistable constraint criterion and single threshold criterion.

Figure S16. Hardware for the speech module in the circuit diagram.

Figure S17. Hardware for the display module in the circuit diagram.

Table S1. Comparison of calculated and recorded minimal angle of motion sensors with

different slit ratios.

Table S2. Comparison of various motion devices and representative device figures of

merit.

Movie S1. Illustration of the hybrid sign language recognition with interactive feedback.


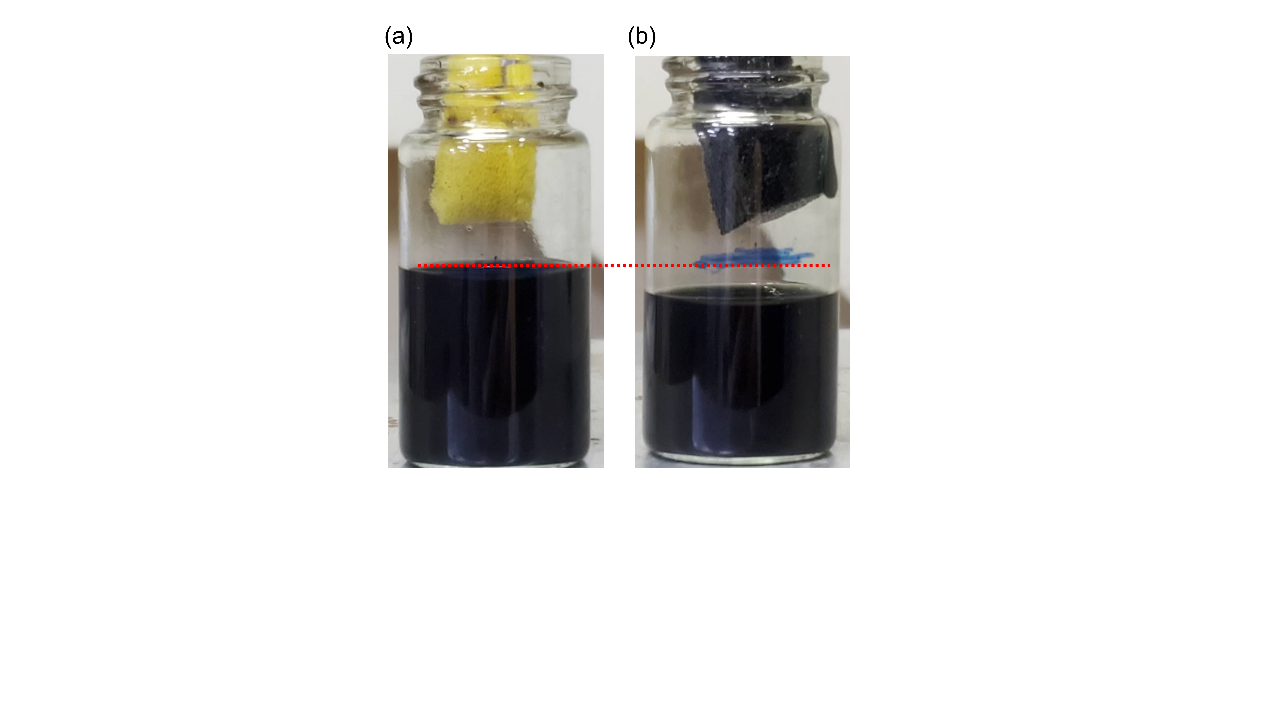


Figure S1: Before and after the one-time dip coating of the PU sponge in the MXene solution.

As shown in Figure S1, the water absorption ability was tested as follows. We cut the PU sponge into cuboids (2 cm in length, 1 cm in width, and 0.5 cm in height). A cuboid was then dipped into the as-prepared MXene aqueous dispersion (3 mg/ml) and squeezed several times to fully absorb the dispersion. 1 ml of MXene aqueous dispersion was absorbed. The water absorption rate of PU sponge could be denoted as:

$R=\frac{V_{water-absorbed}}{V_{PU}}$. (1)

In our test, the water absorption rate could reach 1$ml/cm^{3}$, which indicates the PU sponge is a good candidate for the absorption of coating of MXene.


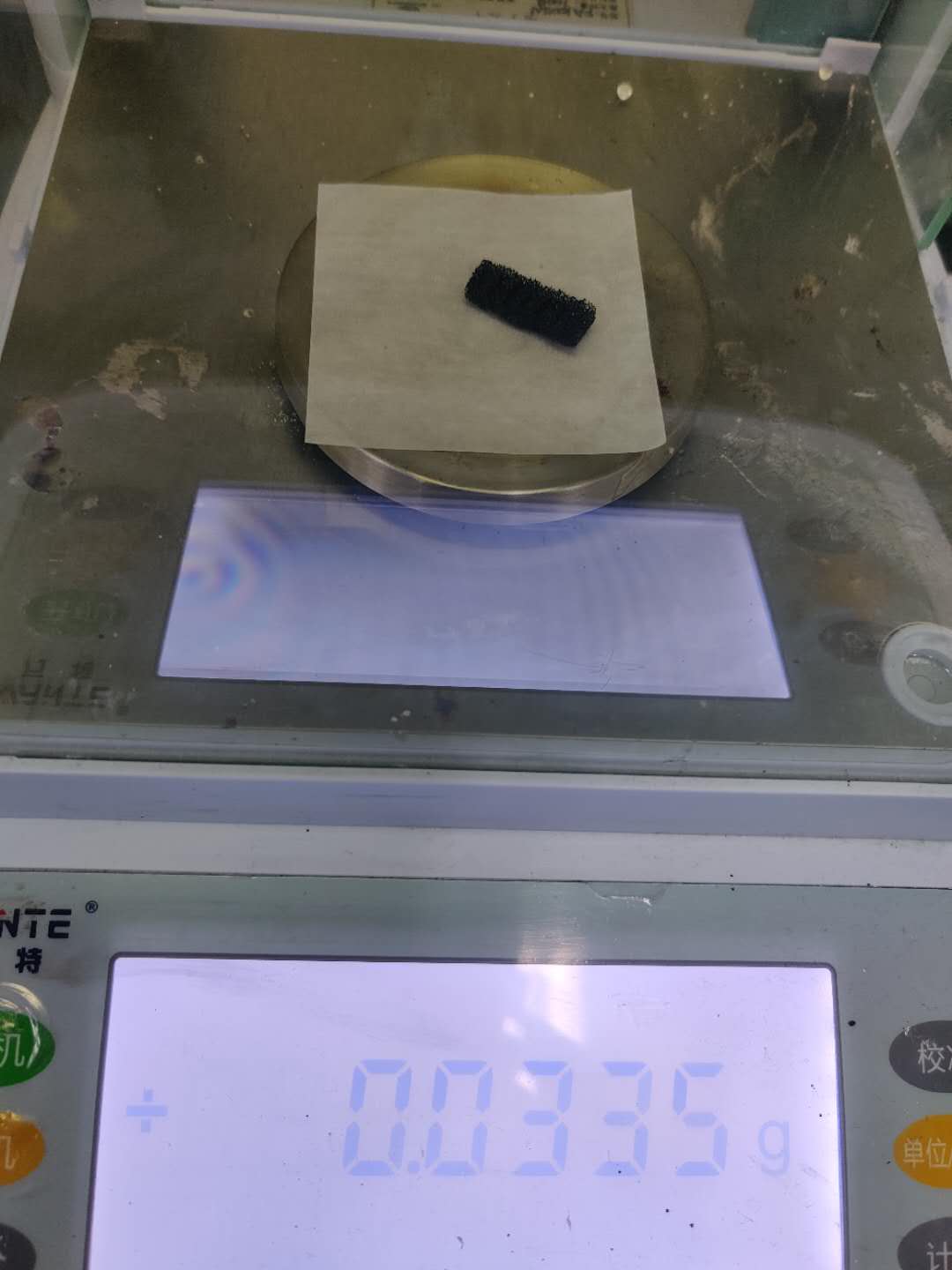


Figure S2: The obtained MXene-coated PU sponge is extremely lightweight, about 33.5 mg.


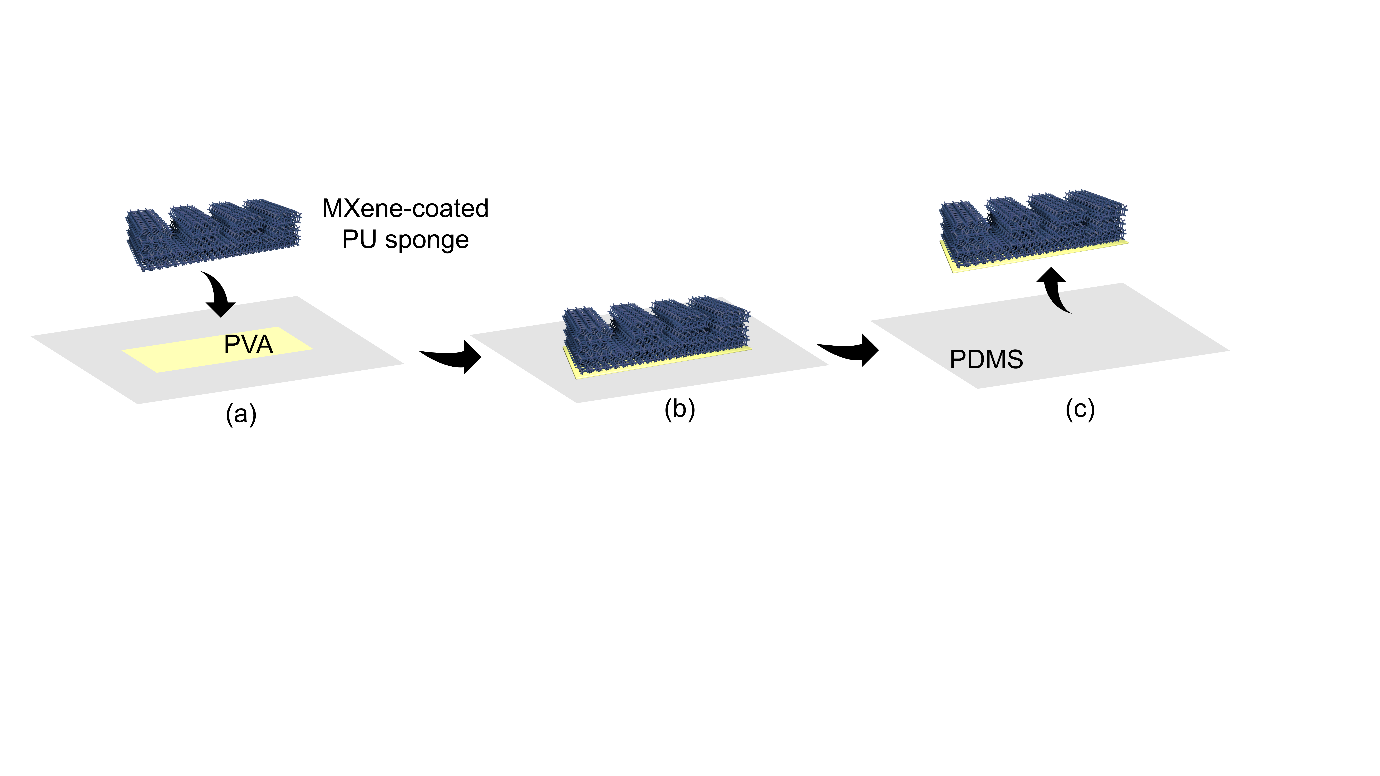


Figure S3**:** The one-step transfer technique to prepare the motion sensor. (a) The gel state PVA was first scraped onto the PDMS substrate uniformly. (b) The as-prepared MXene-coated PU sponge was put onto the PVA gel film and then solidified at room temperature for about 1 h. (c) Finally, the MXene-coated PU sponge with the PVA thin film was peeled off from the PDMS substrate.


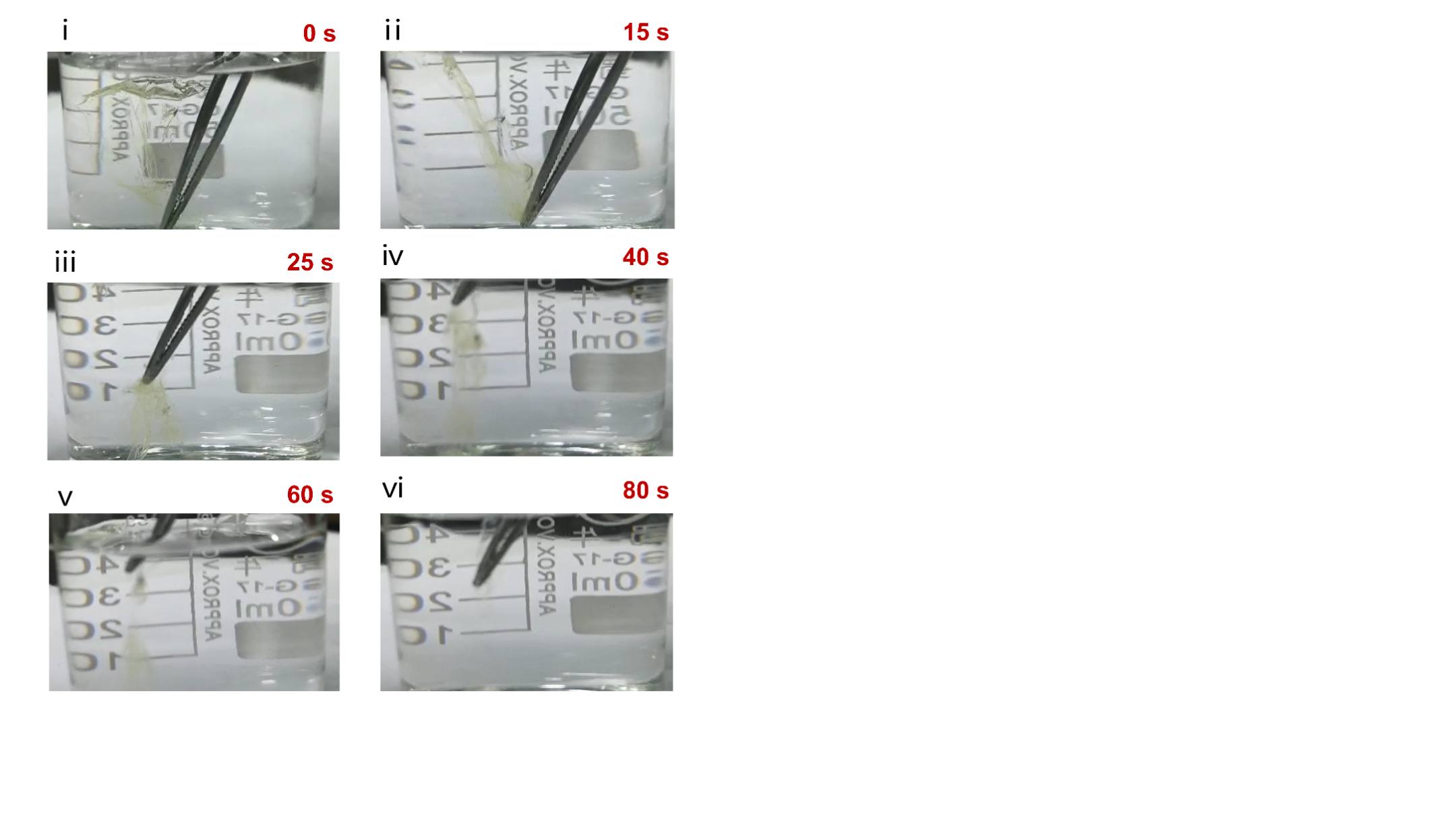


Figure S4: The dissolution of the PVA film in deionized water at room temperature.

The PVA film was dipped into water at room temperature (25 °C). The PVA film began to dissolve after 15 s in water. After 60 s, most parts of the PVA film were dissolved in the deionized water. The PVA film could be completely disposed of after ~80 s. The experiment indicated that the PVA film could be disposed of by simply being in contact with the deionized water. Its biodegradability makes it easy to be completely removed from the skin using water, without any residual, which makes it a good choice to be the buffer layer between the microstructured MXene-coated PU sponge and skin.


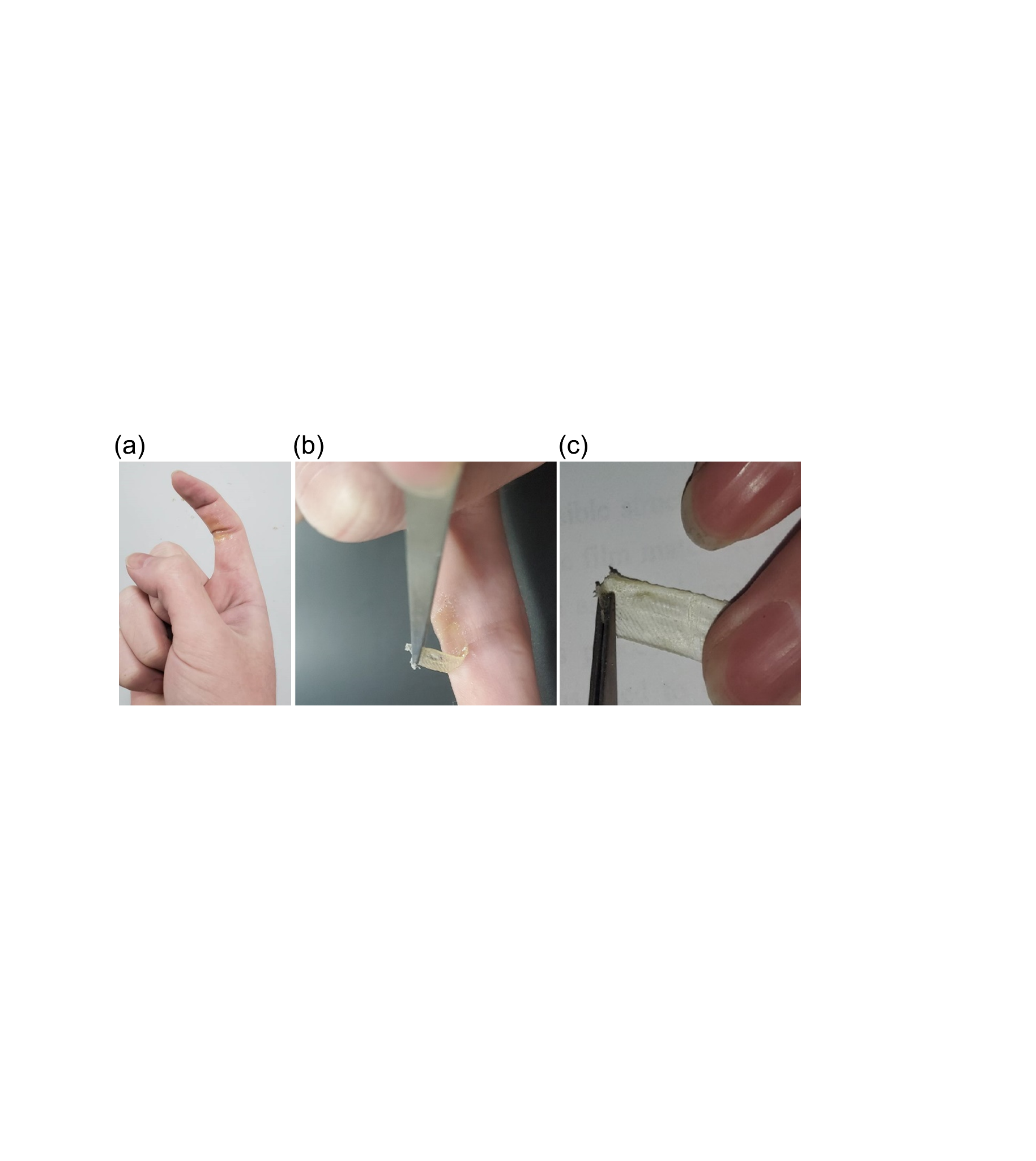


Figure S5: Conformal contact between the PVA and the skin. (a) An ultra conformal contact was formed between the solidified PVA thin film and the skin. (b) The PVA gel thin film is easy to be peeled off from the skin. (c) The PVA film peeled off from the skin retains the texture information of the skin.


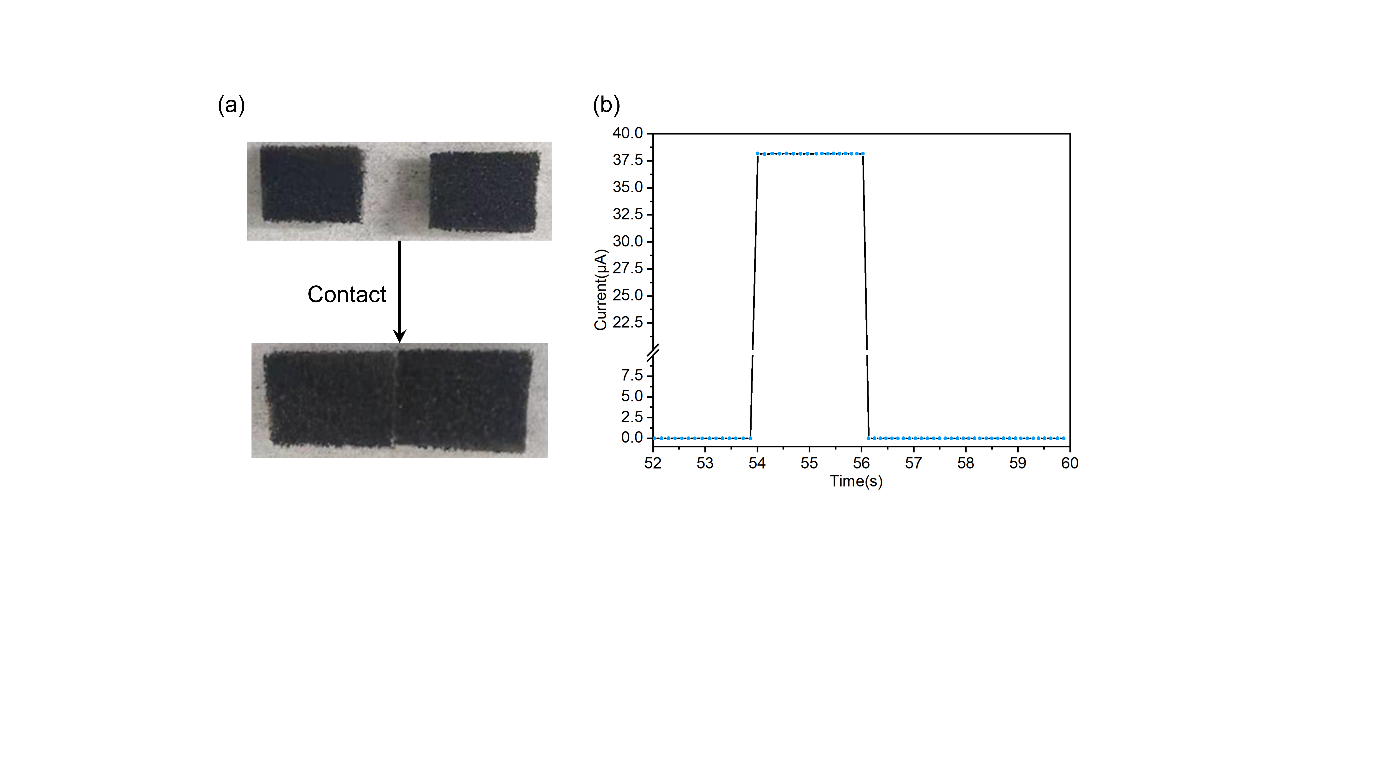


Figure S6: Electrical conductance increases after the two MXene-coated PU blocks contact with each other.


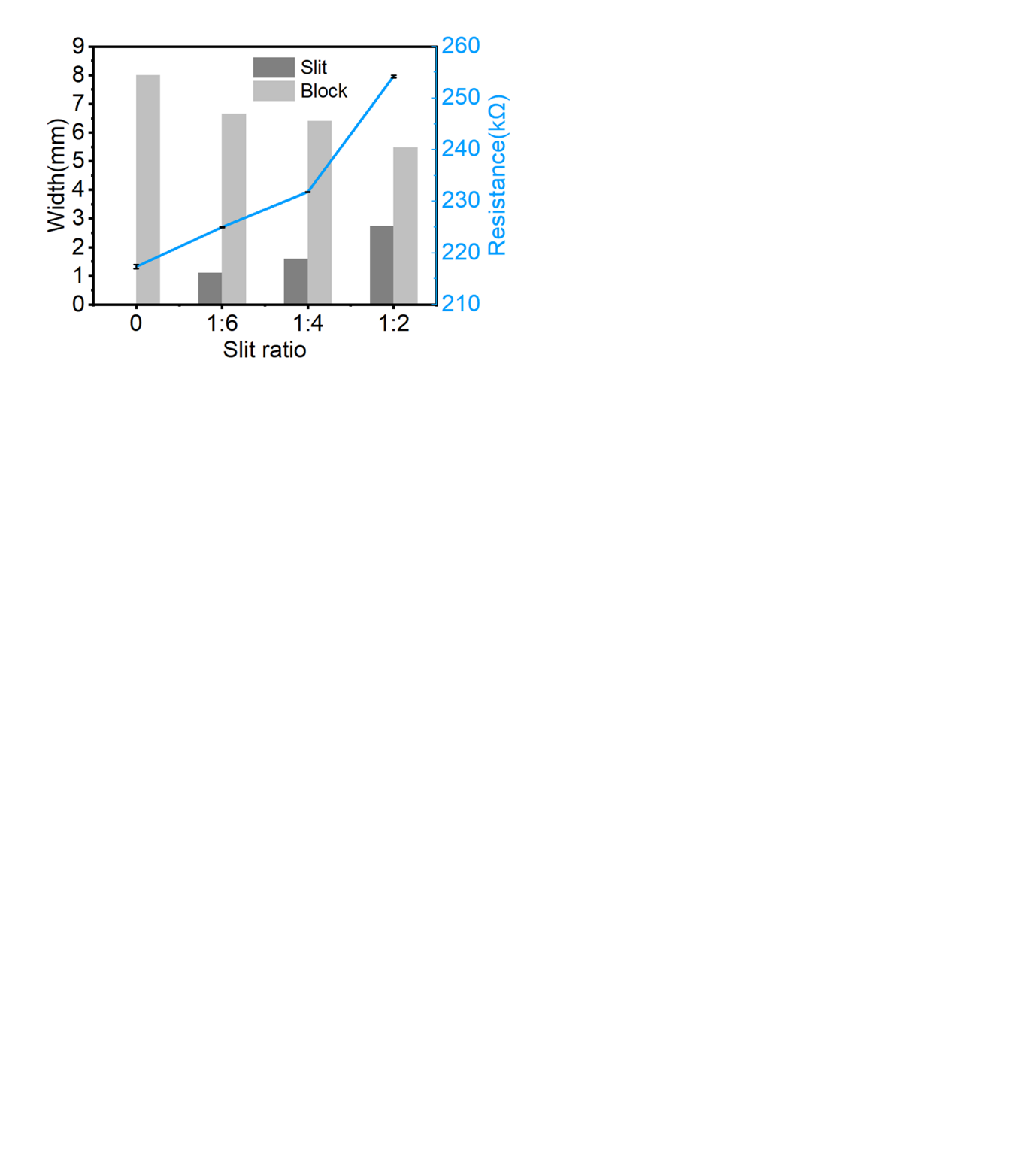


Figure S7. The width of blocks and slits of sensors with different slit ratios and their initial resistances.


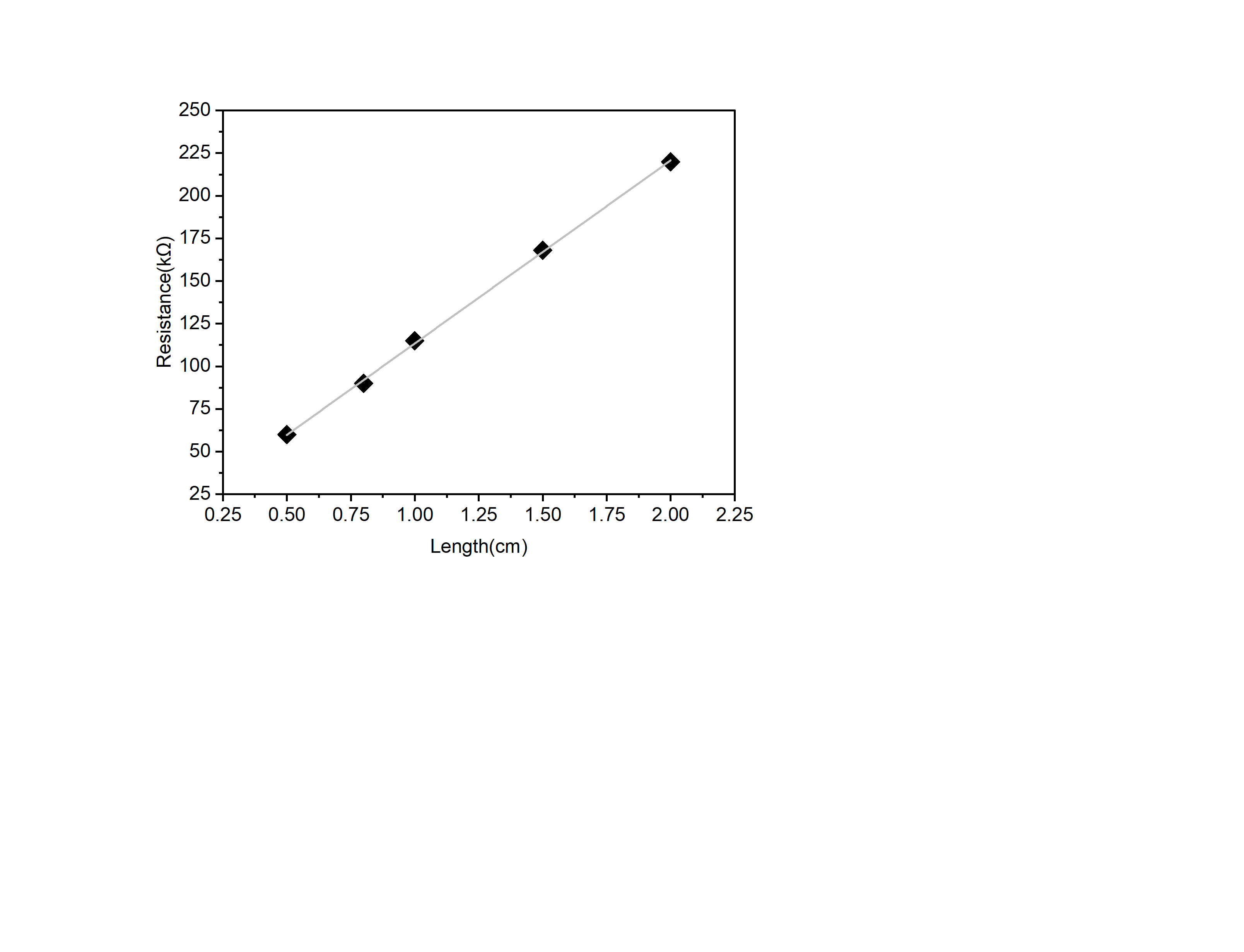


Figure S8: A linear relationship between the initial resistance and sensor length is observed.


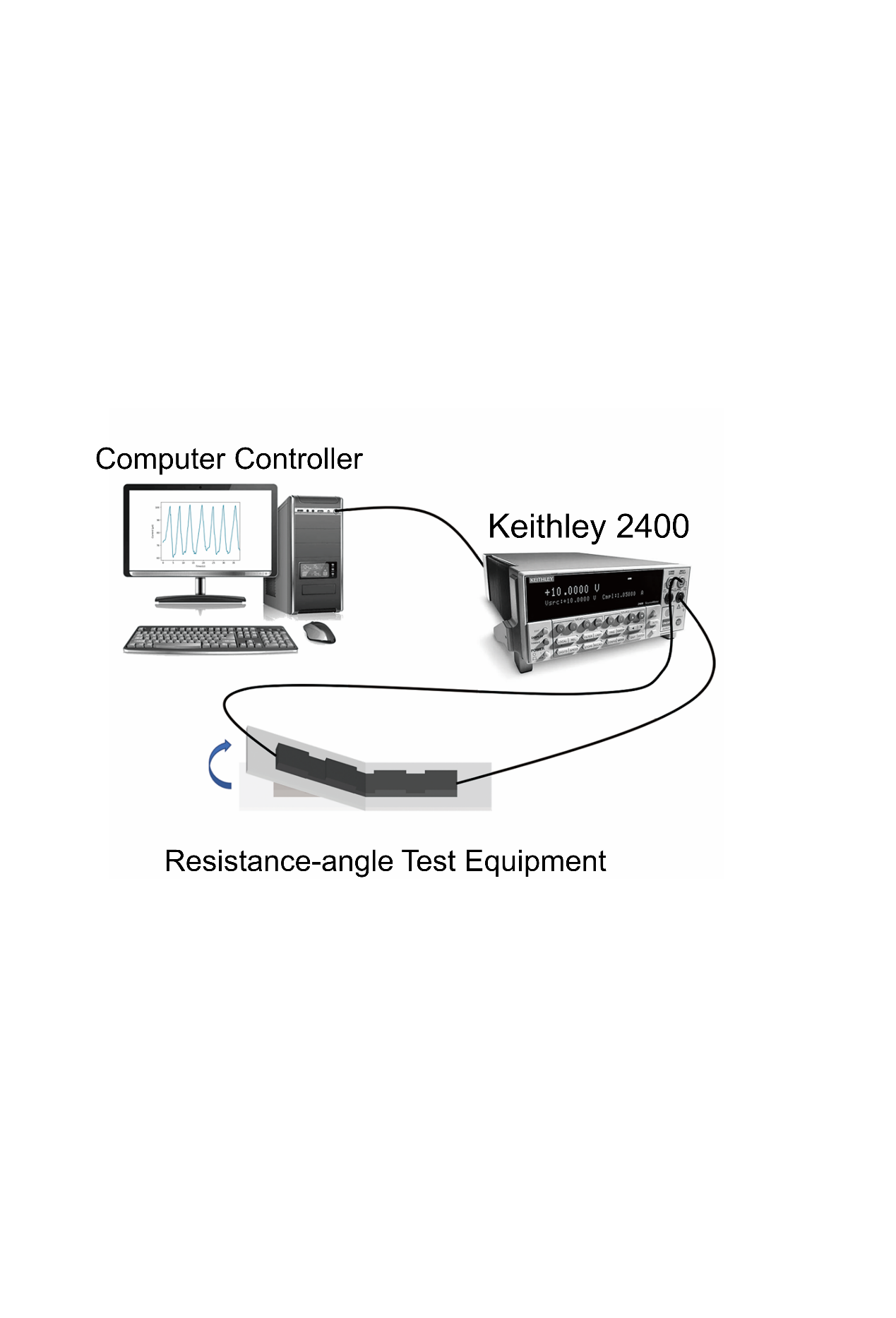


Figure S9: The schematic illustration of the pre-designed testing equipment.

The angle-sensing properties of the motion angle sensor were tested by an electrical signal test system, as shown in the figure. The test system is composed of a computer controller, a source meter (Keithley 2400), and bending equipment. By fixing the motion angle sensor at the bending equipment, the effect of different slit ratios on bending contact effect during bending can be further studied to analyze the angle-sensing performance of motion sensors with different microstructures. The source meter records the current signals of the motion sensor during the bending process and transfers the signals to the computer controller. The computer controller then acquires and processes the signals.


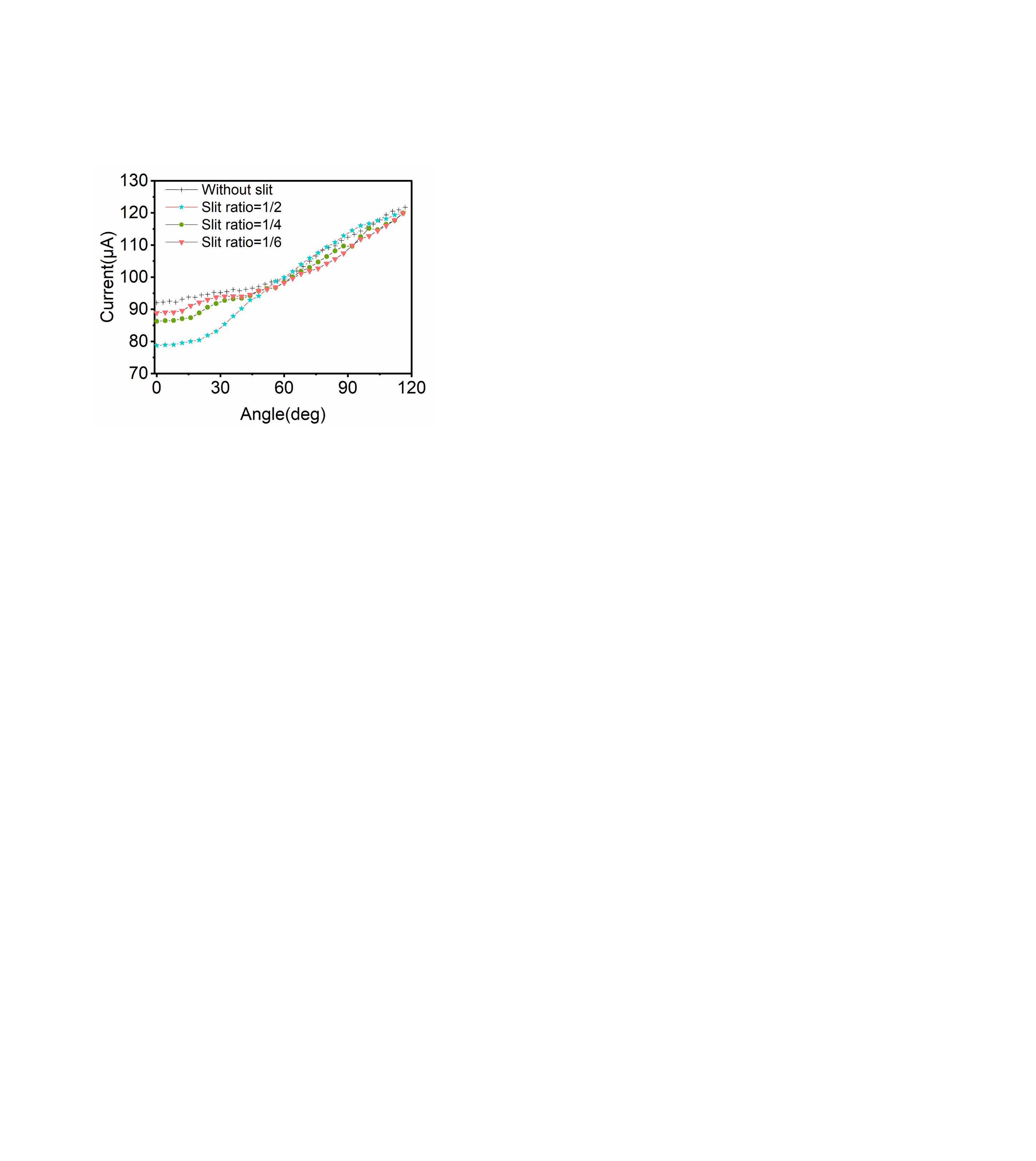


Figure S10. The electrical conductance of sensors with different slit ratios when the

bending angle increases from 0 to 120°.


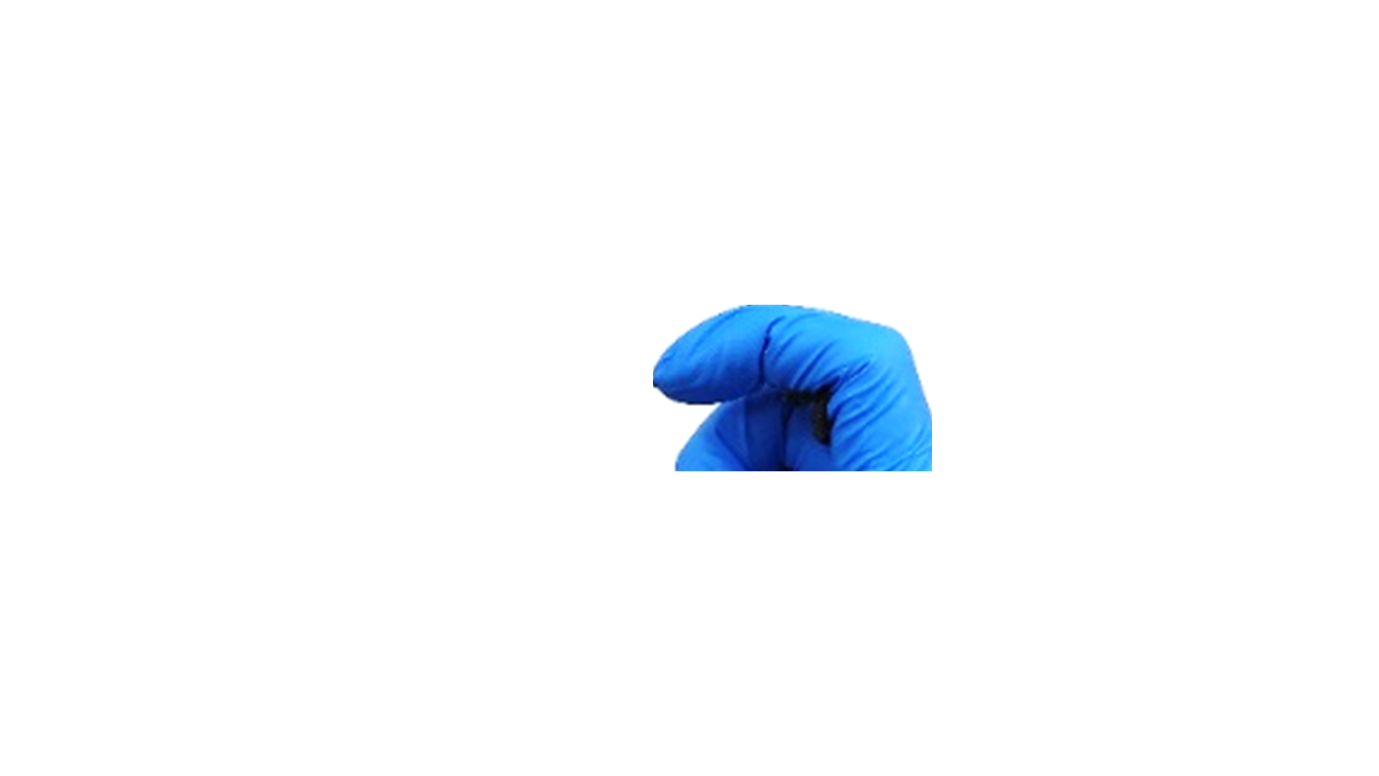


Figure S11: Photograph of motion sensors when the bending angle is up to 90°, where the adjacent PU blocks fully contact with each other.


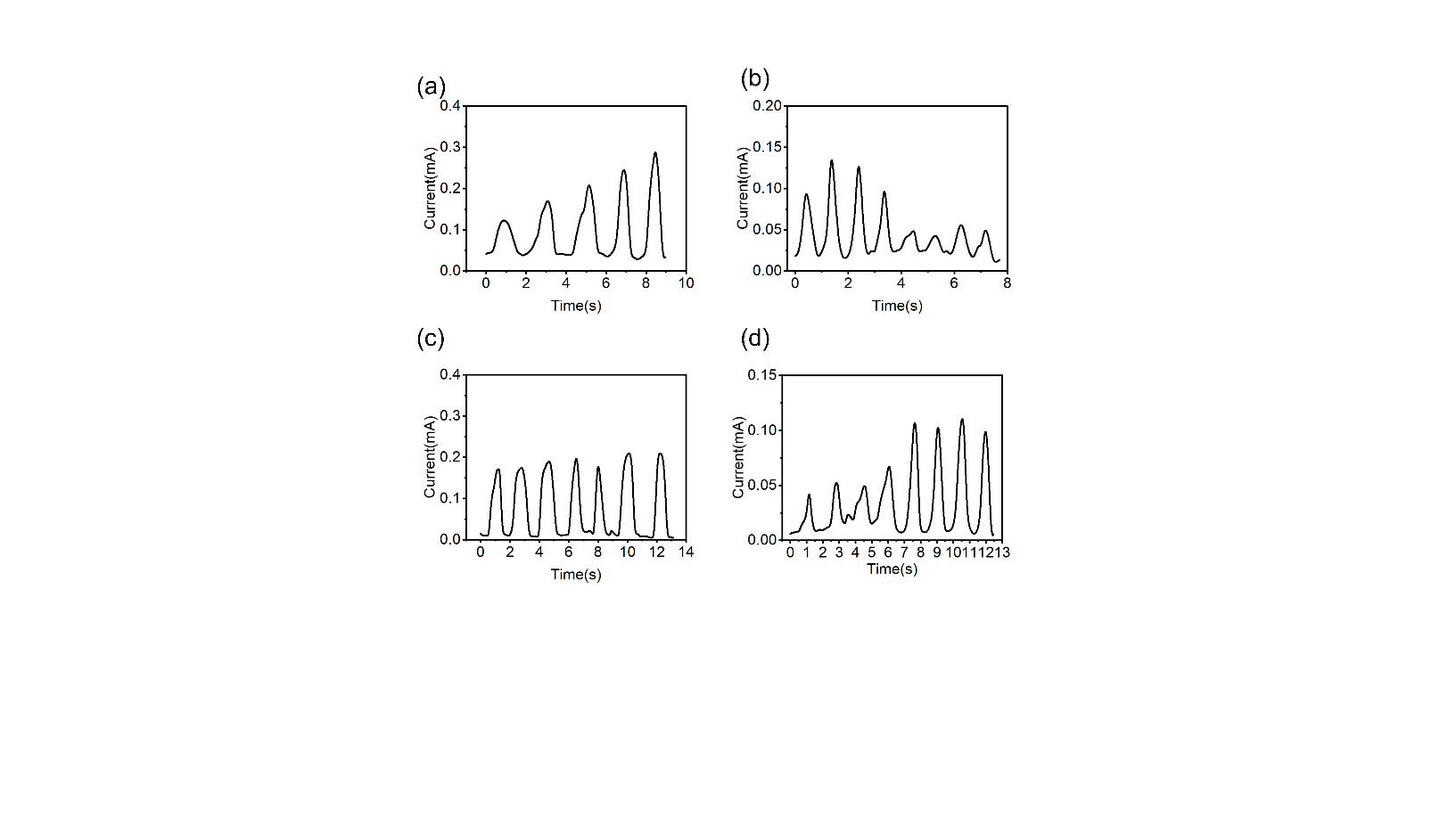


Figure S12: Responses of motion sensors during different joint movements involving (a) wrist, (b) throat, (c) knee, (d) neck. The bending angle of the wrist increases gradually, and the bending angles of the throat and neck are fixed at a small bending angle (75°) and a large bending angle(20°) and the bending angle of the knee is 75°.


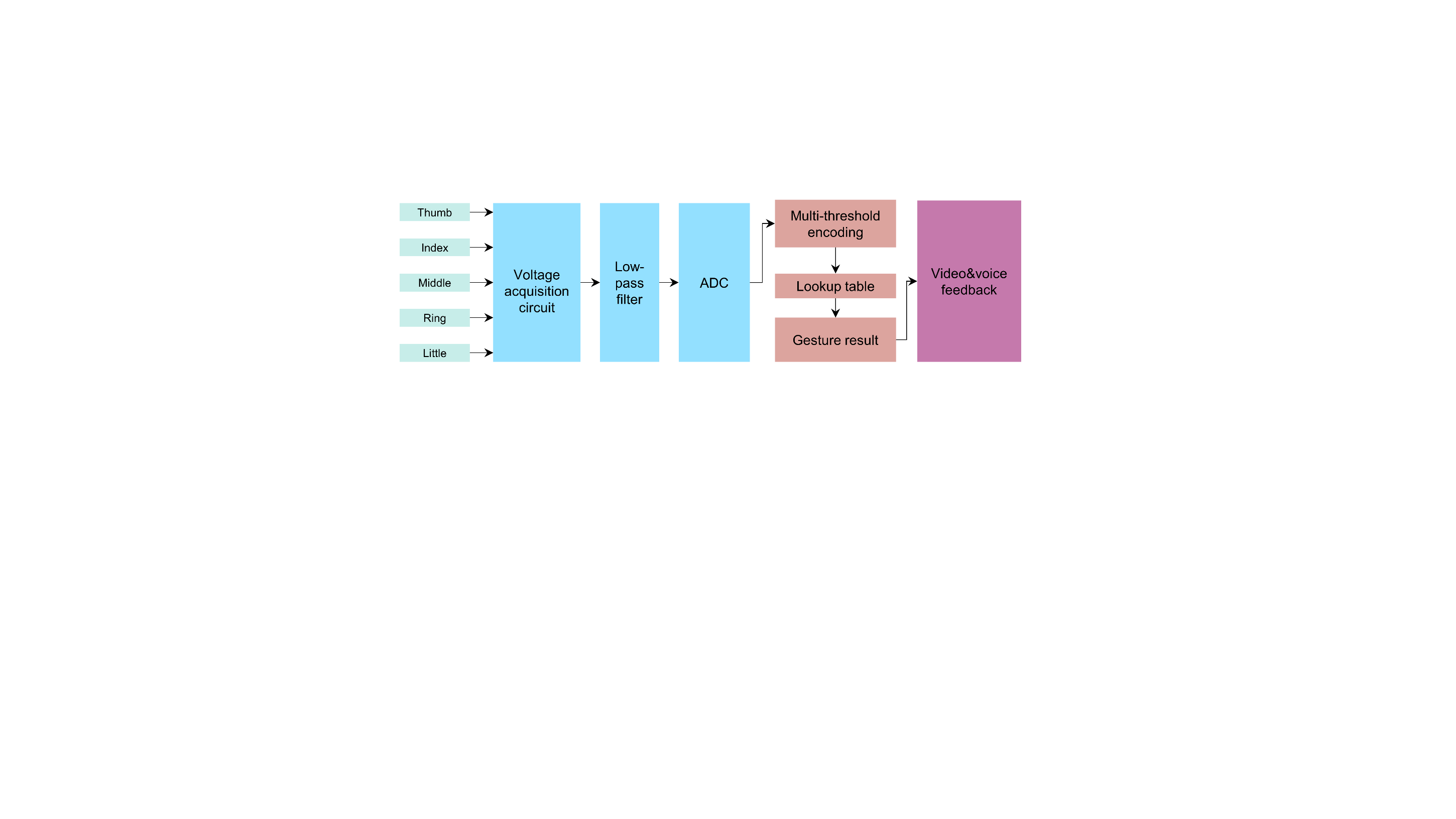


Figure S13: Schematic overview of the hybrid sign language recognition with both hardware and software, beginning with an analog signal acquisition, followed by data processing, and finally, transmission to a customized feedback application.


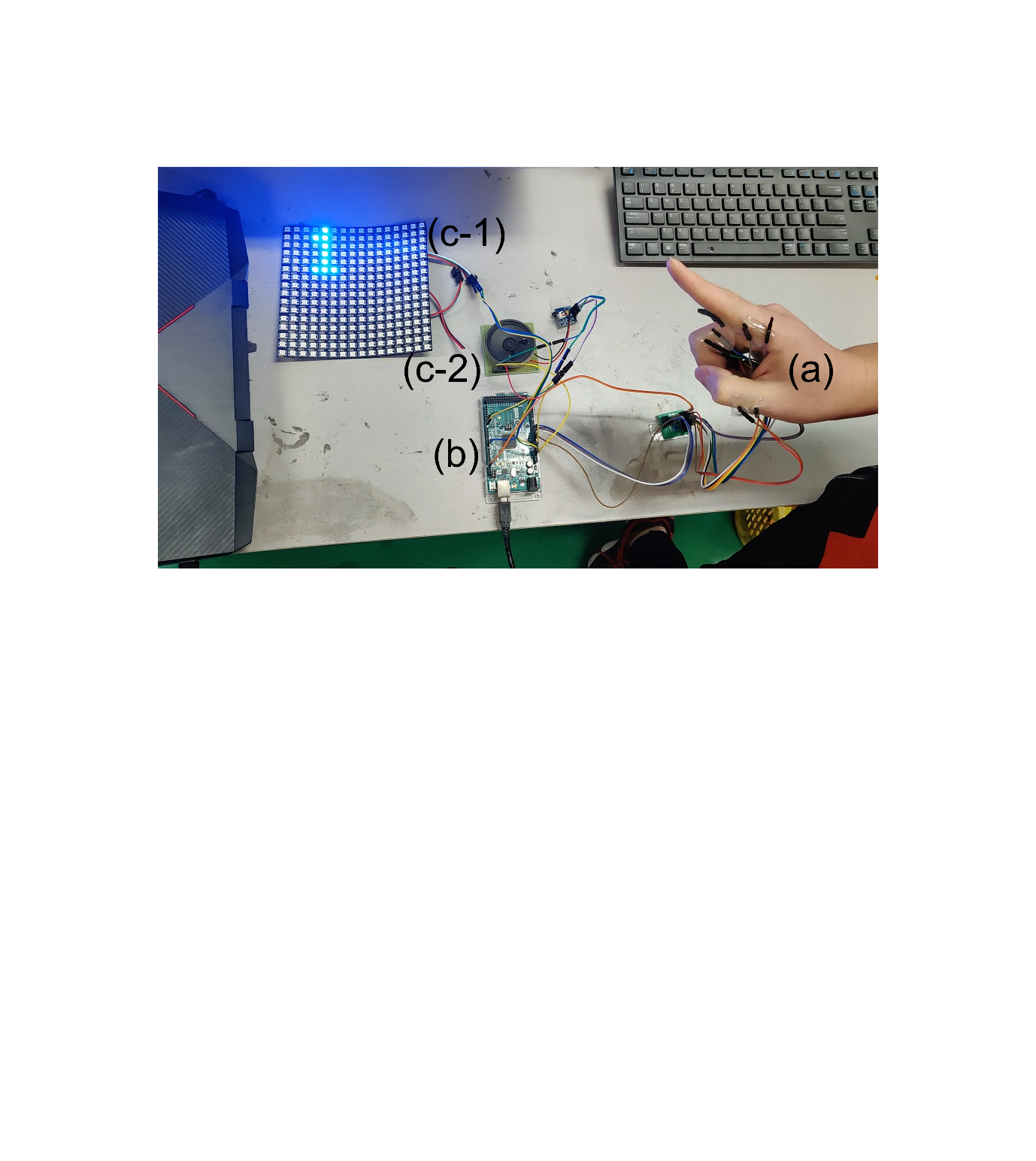


Figure S14: The whole system consists of a wearable sensor array (a), an embedded core (b), and a feedback module including video (c-1) and voice(c-2) feedback.


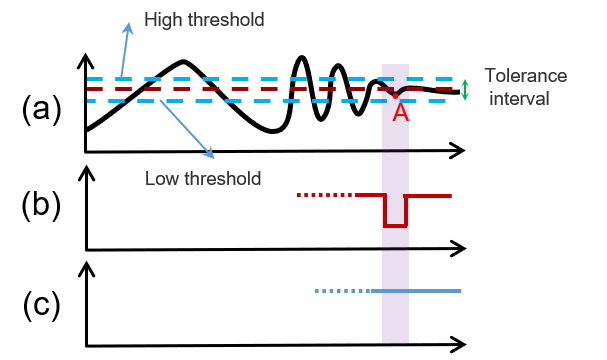


Figure S15. Comparison of the bistable constraint criterion and single threshold criterion.

Referring to the working principle of the Schmitt trigger that encodes the voltage signal depending on high threshold voltage (H_t_) and low threshold voltage (L_t_), we have developed a bistable constraint criterion.

Specifically, the value between the high threshold and the low threshold is denoted as the tolerance interval. When the voltage is between tolerance intervals, the code will be kept at the previous state. For example, for the point, ‘A’, of voltage jitter in (a), traditional single threshold criterion (b) will encode the point ‘A’ as a different code (bending state) and consequently cause encoding errors. However, the bistable encoding system (c) will perfectly avoid this error, which is determined by its unique working principle.


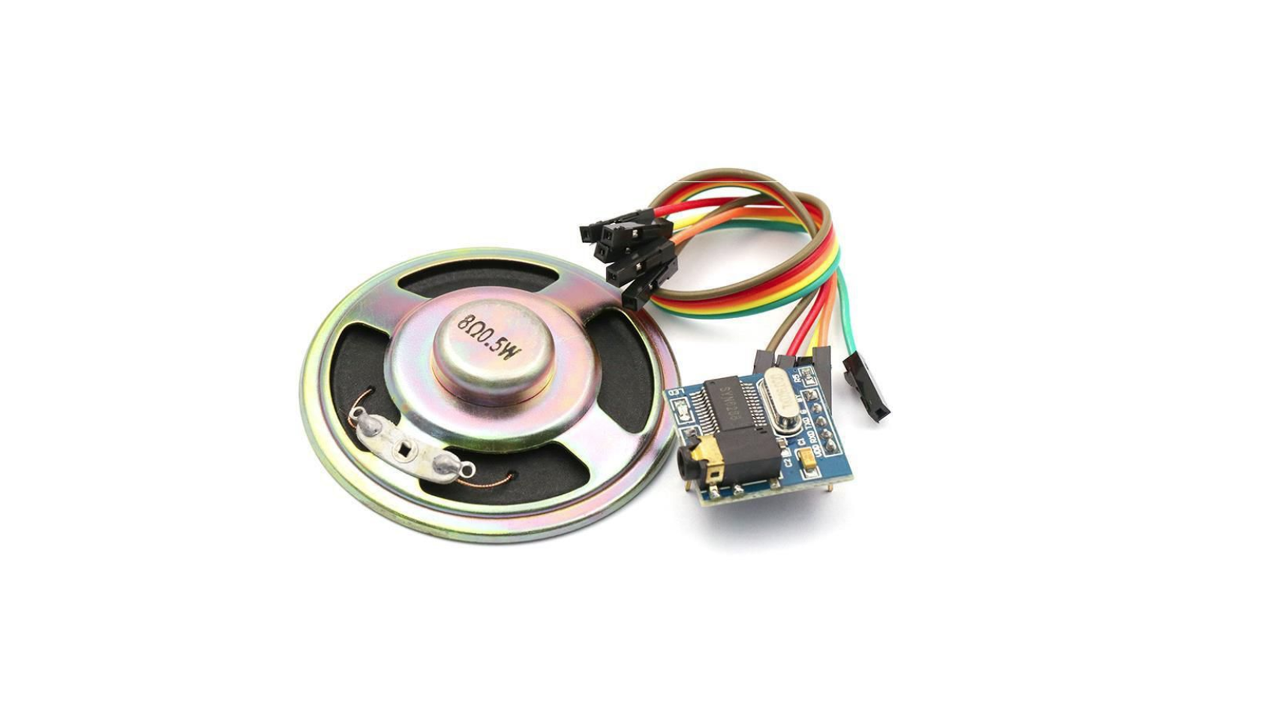


Figure S16. Hardware for the speech module in the circuit diagram. The Text-to-Speech

chip was chosen as the hardware for the speech module. Its built-in algorithm in the chip could do automatic real-time translation of letters into allophone addresses and broadcast them from a loudspeaker. Different Text-to-Speech chips could be found on the website, for example, <https://www.infotalkcorp.com/products/infotalk-speaker/>.


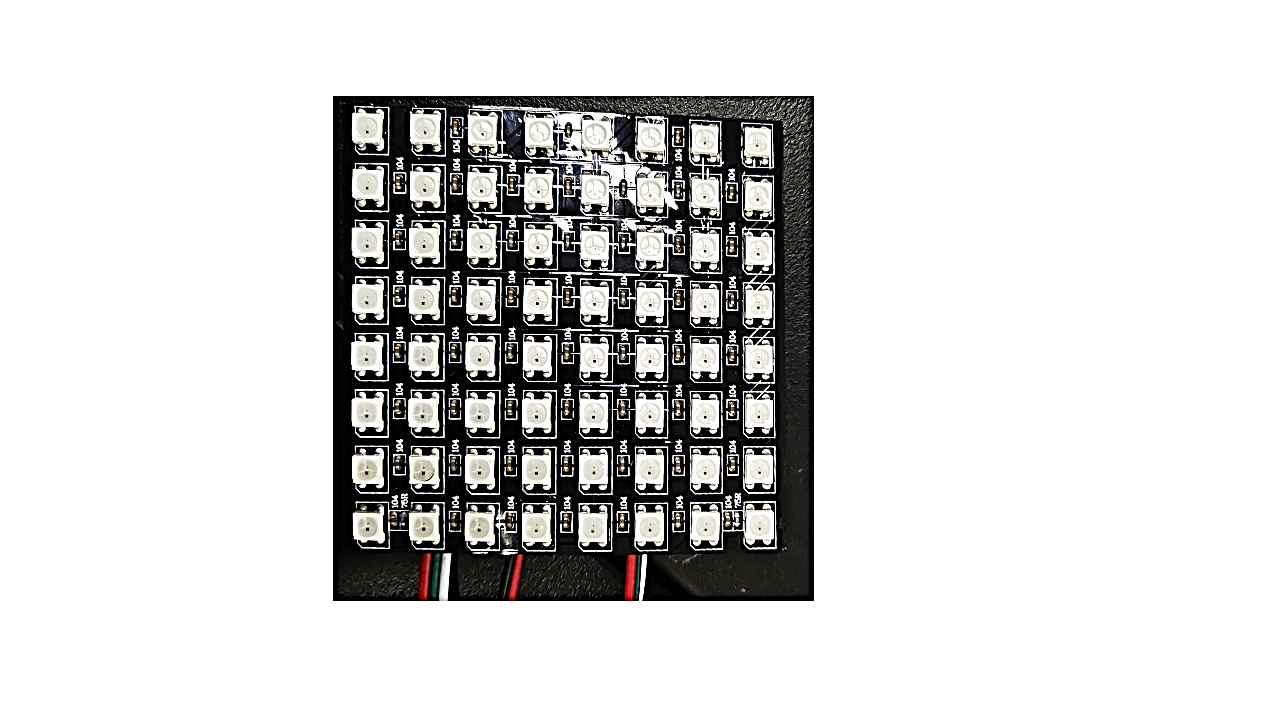


Figure S17: Hardware for the display module in the circuit diagram. The LED lattice screen was chosen as the hardware for the display module owing to its low cost and high customizability in color and pattern for different digits and letters.

Table S1. Comparison of calculated and recorded minimal angle of motion sensors with different slit ratios.

| Slit ratio | S(mm) | Bh(mm) | Calculated minimal angle(°) | Recorded minimal angle(°) |
| --- | --- | --- | --- | --- |
| 1:2 | 1.82 | 3 | 32.7 | ~26 |
| 1:4 | 0.95 | 3 | 19.9 | ~16 |
| 1:6 | 0.74 | 3 | 14.0 | ~12 |

.

Table S2. Comparison of various Motion devices and representative device figures of merit.

| # | Materials | Sensitivity | Range/Strain | Linearity | Type | Reference |
| --- | --- | --- | --- | --- | --- | --- |
| 1 | Al/cellulose paper | 3.23 | Bending strain < 1.67% | No | Antenna | [1] |
| 2 | CNT/PU  elastomer | - | - | No | Capacitive | [2] |
| 3 | Cu/FEP | Minimum  resolution  angle:  3.8° | - | No | Triboelectric | [3] |
| 4 | Au/PMMA | - | - | Yes | Resistive | [4] |
| 5 | Ag/Kapton | - | 15° -45° | No | Triboelectric | [5] |
| 6 | PVDF/ZnO | 4.4mV/deg | 44° -122° | No | Piezoelectric | [6] |
| 7 | Mxene/PU | ~0.45%/deg | 15°-120° | Yes | Resistive | This work |

1. Kanaparthi, S.; Sekhar, V. R.; Badhulika, S. Flexible, eco-friendly and highly sensitive paper antenna based electromechanical sensor for wireless human motion detection and structural health monitoring. Extreme Mech. Lett. **2016,** 324-330.

2. Nakamoto, H.; Ootaka, H.; Tada, M.; Hirata, I.; Kobayashi, F.; Kojima, F. Stretchable Strain Sensor With Anisotropy and Application for Joint Angle Measurement. IEEE Sens. J. **2016,** 3572-3579.

3. Pu, X.; Guo, H.; Tang, Q.; Chen, J.; Feng, L.; Liu, G.; Wang, X.; Xi, Y.; Hu, C.; Wang, Z. L. Rotation sensing and gesture control of a robot joint via triboelectric quantization sensor. Nano Energy **2018,** 453-460.

4. Haiting, W.; Yanhong, T.; Xiaoli, Z.; Qingxin, T.; Yichun, L. Flexible, high-sensitive, and wearable strain sensor based on organic crystal for human motion detection. Org. Electron. **2018,** 304-311.

5. Wang, S.; He, M.; Weng, B.; Gan, L.; Zhao, Y.; Li, N.; Xie, Y. J. N. Stretchable and wearable triboelectric nanogenerator based on kinesio tape for self-powered human motion sensing. Nanomaterials **2018,** 657.

6. Deng, W.; Yang, T.; Jin, L.; Yan, C.; Huang, H.; Chu, X.; Wang, Z.; Xiong, D.; Tian, G.; Gao, Y.; Zhang, H.; Yang, W. Cowpea-structured PVDF/ZnO nanofibers based flexible self-powered piezoelectric bending motion sensor towards remote control of gestures. Nano Energy **2019,** 516-525.
